# Supplementary material for: Observable variations in human sex ratio at birth
Source: PLoS Comput Biol. 2021 Dec 2;17(12):e1009586. doi: 10.1371/journal.pcbi.1009586 (PMC8638995; doi:10.1371/journal.pcbi.1009586)
Supplement: S1 Appendix — Fig A. Distribution of the SRB in the US and Sweden at the county level (US) or the kommun level (Sweden). Fig B. Dendrogram with statistically significant clusters (95% level) in red boxes. Table A. Differences in information criterion (ΔIC) and their standard errors (SE) of individual factors with fixed-effect only. Non-significant factors are omitted. Table B. Differences in information criterion (ΔIC) and their standard errors (SE) of individual factors with fixed-effect only. Non-significant factors are omitted. Fig C. Time series plots and out-of-sample forecasts for SRB data grouped into 28-day periods and fitted with seasonal ARIMA models. The blue shade is the 95% confidence level. The observed SRBs for the first 5 months after the intervention are presented by red dots, whereas the observed SRBs for 7–9 months after the intervention are presented by purple dots. See also Table C. Table C. Out-of-sample forecasts for the first 10 months after the intervention using SRB data grouped into 28-day periods and fitted with seasonal ARIMA models. Any period of which the observed SRB is outside of the 95% confidence level is marked by an asterisk (*). Figure D. Time series plots and out-of-sample forecasts for SRB data grouped into 28-day periods and fitted with state-space models. The blue shade is the 95% confidence level. The observed SRBs for the first 5 months after the intervention are presented by red dots, whereas the observed SRBs for 7–9 months after the intervention are presented by purple dots. See also Table D. Table D. Out-of-sample forecasts for the first 10 months after the intervention using SRB data grouped into 7-day periods and fitted with state-space models. Any period of which the observed SRB is outside of the 95% confidence level is marked by an asterisk (*). Fig E. Time series plots and out-of-sample forecasts for SRB data grouped into 28-day periods and fitted with seasonal ARIMA models. The blue shade is the 95% confidence level. The obse [file pcbi.1009586.s001.pdf]

Supplementary Information for *Observable Variations in Human Sex Ratio at Birth*

Yanan Long[1,2,3], Qi Chen[4], Henrik Larsson[4,5], and Andrey Rzhetsky[2,3,6]

[1]Department of Chemistry, The University of Chicago, Chicago, Illinois, United States of America
[2]Department of Medicine, The University of Chicago, Chicago, Illinois, United States of America
[3]Institute of Genomics and Systems Biology, The University of Chicago, Chicago
[4]Department of Medical Epidemiology and Biostatistics, Karolinska Institutet, Stockholm, Sweden
[5]School of Medical Sciences, Örebro University, Örebro, Sweden
[6] Department of Human Genetics and Committee on Quantitative Methods in Social, Behavioral, and Health Sciences, The University of Chicago, Chicago, Illinois, United States of America

I Overall SRB Distribution

Figure A shows that the distributions of sex ratio at the county level (USA) or the kommun level (Sweden) are very similar, with the US having an overall SRB of 0.5142 and Sweden 0.5139.

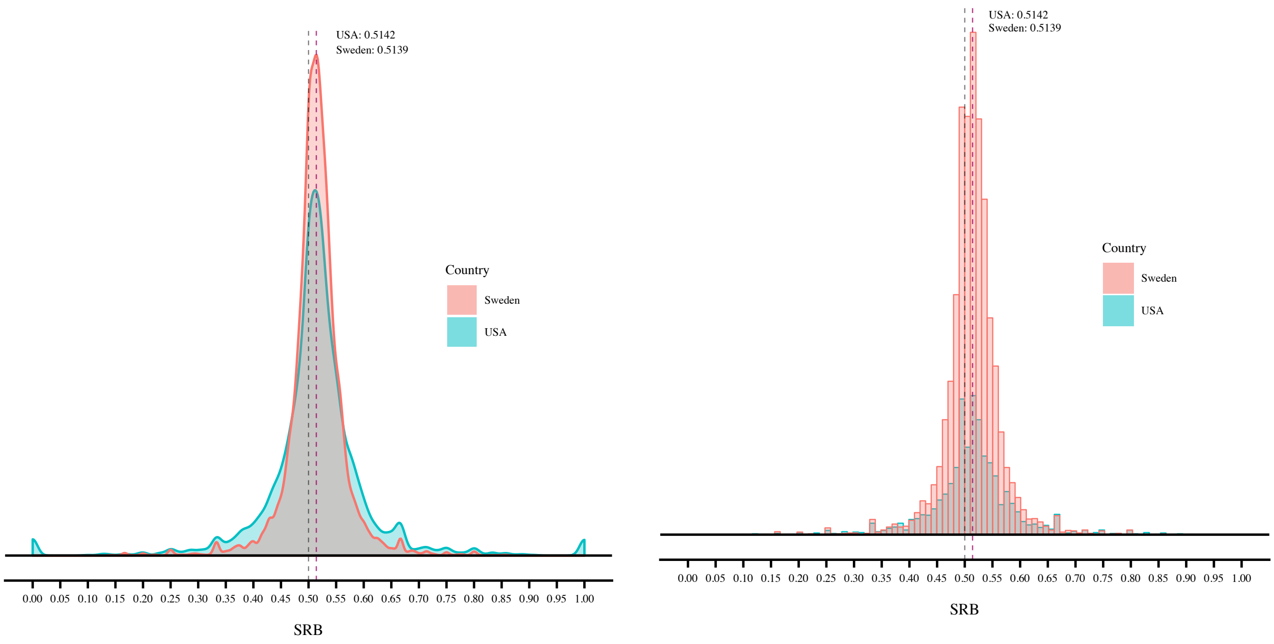

(a) Kernel density estimation (KDE)

(b) Histogram

Figure A: Distribution of the SRB in the US and Sweden at the county level (US) or the kommun level (Sweden)

II Cluster Analysis

Figure B shows the dendrogram of the clustering the factors in the US EQI data set by Ward's method (see the Methods section in the main text for more detail). Each red box delimits a statistically significant cluster (at the 95% level), which contains at least 2 factors.

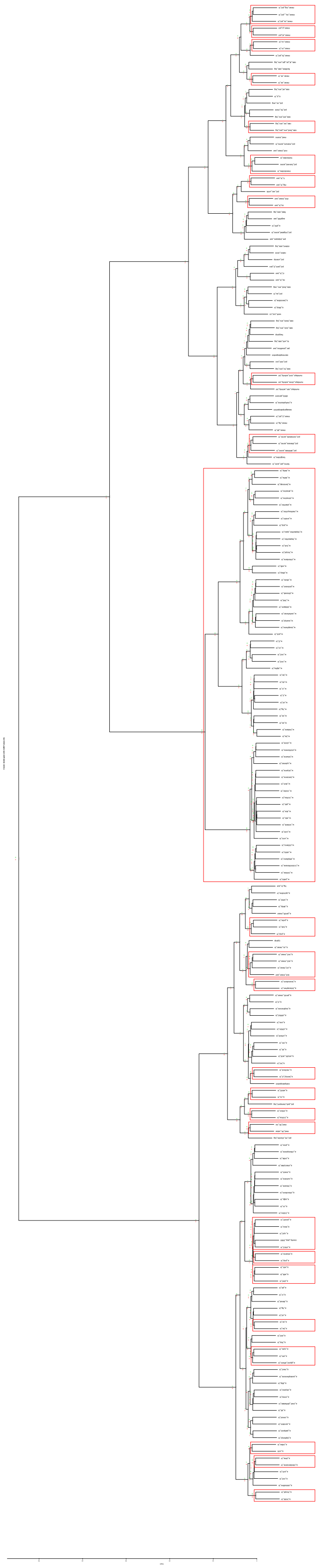

Figure B: Dendrogram with statistically significant clusters (95% level) in red boxes

III Regression

Tables A and B, respectively, list all the statistically significant factors (8 for fixed-effect and all 125 for mixed-effect), sorted by decreasing ΔIC, with respect to the null.

Factor	ΔIC	SE
cluster_ward_8	25.6880	7.7901
cluster_ward_1	21.5759	6.4210
cluster_ward_11	20.9930	5.1878
cluster_ward_15	20.8578	4.1703
sewagenpdesperkm	19.0802	4.0902
pct_no_eng	16.1841	4.2216
a_dbp_ln	13.9700	3.8312
pct_mt_10units_log	10.5599	3.3704

Table A: Differences in information criterion (ΔIC) and their standard errors (SE) of individual factors with fixed-effect only. Non-significant factors are omitted.

Table B: Differences in information criterion (ΔIC) and their standard errors (SE) of individual factors with the random effect at the state level in the US EQI dataset.

Factor	ΔIC	SE
pct_rent_occ	54.2185	8.7760
cluster_ward_9	52.3907	8.8256
mean_pb_ln	51.7339	9.6749
farms_per_acre_ln	51.6266	9.0878
cluster_ward_15	51.5511	8.5623
pct_mt_10units_log	51.3259	8.5483
rate_food_env_neg	51.3230	9.2907
a_isophorone_ln	50.9430	10.3995
rate_ent_env_log	50.9259	8.8088
a_dbp_ln	50.4537	8.6102
a_mn_ln	50.2819	8.1831
hg_ln_ave	50.0576	8.6584
ryprop	49.6911	9.4066
a_n2h2_ln	49.5381	8.4877
pct_vac_units	49.3265	8.5257
fatal_rate_log	49.0348	8.3392
cluster_ward_25	48.6212	9.8838
indnpdesperkm	48.5428	10.2509
a_biphenyl_ln	48.4603	8.6128
a_cn_ln	48.1418	9.7888
cluster_ward_10	47.7198	8.7054
work_out_co	47.4916	8.9555
mean_fe_pct_ln	47.2117	8.0750
cluster_ward_19	47.1722	8.5983
cluster_ward_26	47.1691	8.7901
a_quinoline_ln	47.1477	9.6906
a_sb_ln	47.0255	8.3046
cluster_ward_8	46.3741	8.9328

Continued on next page

Table B: Differences in information criterion (ΔIC) and their standard errors (SE) of individual factors with the random effect at the state level in the US EQI dataset.

Factor	ΔIC	SE
med_rooms	46.3220	8.1562
rate_al_pn_gm_env_log	46.3080	8.3765
no3_mean_ave	46.1843	8.8100
a_meoh_ln	45.9854	8.3145
cluster_ward_6	45.9191	8.6963
d303_percent	45.8835	9.4942
na_ln_ave	45.0601	8.1385
a_co_mean_ln	44.9827	8.5933
a_c6h5cl_ln	44.6342	8.7028
a_me2_phthalate_ln	44.4614	8.0751
a_acrylic_acid_ln	44.2292	9.8383
a_mecl_ln	44.0207	8.4892
cat	43.9278	8.3540
mean_ti_pct_ln	43.8613	8.8678
cluster_ward_18	43.8523	9.3605
a_c2hcl3_ln	43.7136	8.7911
a_pahpom_ln	43.6743	9.1561
a_benzidine_ln	43.6200	8.4935
rate_civic_env_log	43.6027	8.5611
fungicides_ln	43.5278	8.7614
cluster_ward_3	43.5188	8.9542
pct_irrigated_acres_ln	43.4427	9.8728
a_p_ln	43.2101	8.2484
to_unit_rate_log	42.9314	8.5853
cluster_ward_2	42.8250	9.4466
pct_au_ln	42.8249	9.4111
cluster_ward_11	42.8243	9.0778
a_c3h3n_ln	42.8093	8.4624
a_diesel_ln	42.7350	8.1946
a_etacrylate_ln	42.7323	8.7796
avgofd3_ave	42.5189	8.8904
pct_pub_transport_log	42.1074	8.4142
a_pb_ln	41.9983	8.0841
a_ph3_ln	41.9038	9.0259
a_eox_ln	41.8434	8.9989
pct_unemp	41.8191	7.7675
a_hexane_ln	41.8044	8.0627
pct_no_eng	41.7541	8.2477
a_acrolein_ln	41.6847	8.2058
a_ech_ln	41.6194	8.4322
a_cumene_ln	41.6100	8.2629
a_11dce_ln	41.5146	8.4598
radon_zone	41.4107	8.6552
a_2np_ln	41.2805	9.3701
a_cr_ln	41.1006	8.3650
cluster_ward_13	41.0139	8.1444
a_pm10_mean_ln	40.8277	8.0920
a_cresol_ln	40.7598	8.2679
rate_trans_env_log	40.7569	8.4773
a_proo_ln	40.7404	8.1918

Continued on next page

Table B: Differences in information criterion (ΔIC) and their standard errors (SE) of individual factors with the random effect at the state level in the US EQI dataset.

Factor	ΔIC	SE
per_totpopss_ave	40.6453	8.2117
a_cl_ln	40.6386	8.2518
stormnpdesperkm	40.5914	8.4561
facilities_rate_log	40.5648	9.2041
pct_manure_acres_ln	40.4699	8.8810
cluster_ward_12	40.4274	9.1367
a_mibk_ln	40.3786	8.7446
a_dehp_ln	40.3305	8.1627
numdays_rain_activity_tot	40.1392	8.3155
cluster_ward_20	39.8644	8.6439
cl_ln_ave	39.5243	9.2021
a_egly_ln	39.4799	8.2135
mean_hg_ln	39.4692	8.1523
rate_ed_env_log	39.3155	8.7520
a_pm25_mean	39.1712	8.5654
cluster_ward_22	39.1668	8.3645
a_hg_ln	39.0513	7.8800
cluster_ward_5	39.0485	8.1282
a_chloroform_ln	38.9772	8.1250
a_cs2_ln	38.4509	7.6905
a_tdi_ln	38.4377	8.9270
per_pswithsw_ave	38.3795	8.1011
a_acetophenone_ln	37.9548	8.6551
violent_rate_log	37.8928	8.8251
a_cd_ln	37.8764	7.9860
a_dbcp_ln	37.7606	8.3128
a_etcl_ln	37.6943	8.3588
a_chloroprene_ln	37.6642	8.4969
cluster_ward_14	37.4994	8.6078
hwyprop	37.3042	8.4589
a_stryene_ln	37.2645	8.3137
cluster_ward_4	37.0974	8.2277
cluster_ward_1	36.9302	8.3875
cluster_ward_16	36.9113	8.0581
cluster_ward_7	36.6871	7.8672
rate_hc_env_log	36.6085	8.1125
cluster_ward_23	36.5280	8.1485
sewagenpdesperkm	36.0821	7.4545
cluster_ward_21	35.9375	8.7625
a_otoluidine_ln	35.8421	7.9246
a_mma_ln	35.3633	8.1445
a_mehydrazine_ln	35.1223	7.4720
cluster_ward_24	35.0831	8.0009
pct_pers_lt_pov	34.9108	7.1347
pct_hs_more	34.8201	8.0671
cluster_ward_17	34.5813	7.9217
a_hcl_ln	34.1790	8.0501

IV Time Series Forecasts

IV.1 Monthly

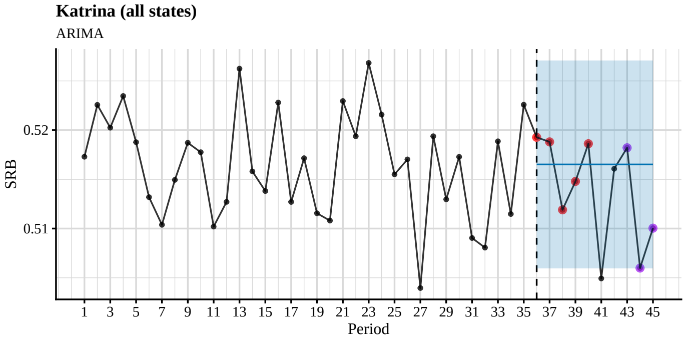

(a) Hurricane Katrina, all states

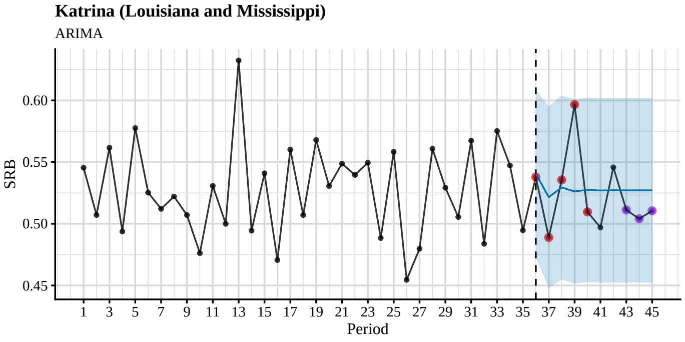

(b) Hurricane Katrina, Louisiana and Mississippi only

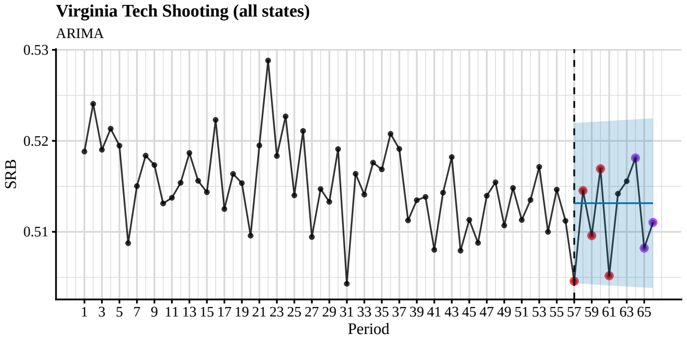

(c) Virginia Tech shooting, all states

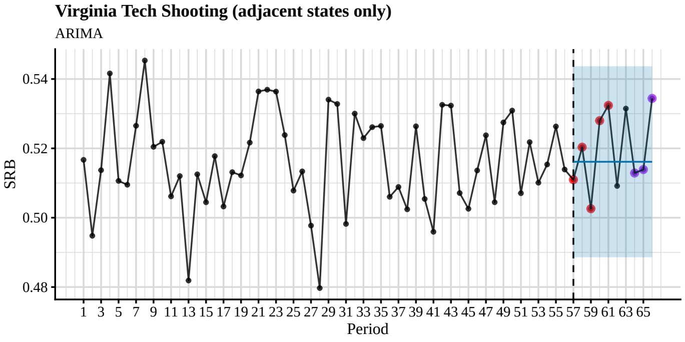

(d) Virginia Tech shooting, adjacent states only

Figure C: Time series plots and out-of-sample forecasts for SRB data grouped into 28-day periods and fitted with seasonal ARIMA models. The blue shade is the 95% confidence level. The observed SRBs for the first 5 months after the intervention are presented by red dots, whereas the observed SRBs for 7–9 months after the intervention are presented by purple dots. See also Table C.

Period	SRB	Lower 95%	Upper 95%
36	0.5193	0.5060	0.5271
37	0.5188	0.5060	0.5271
38	0.5119	0.5060	0.5271
39	0.5148	0.5060	0.5271
40	0.5186	0.5060	0.5271
41(*)	0.5049	0.5060	0.5271
42	0.5161	0.5060	0.5271
43	0.5182	0.5060	0.5271
44	0.5060	0.5060	0.5271
45	0.5100	0.5060	0.5271

(a) Hurricane Katrina in all states

Period	SRB	Lower 95%	Upper 95%
36	0.5379	0.4726	0.6086
37	0.4889	0.4479	0.5952
38	0.5356	0.4549	0.6040
39	0.5966	0.4515	0.6009
40	0.5096	0.4528	0.6023
41	0.4970	0.4522	0.6017
42	0.5458	0.4525	0.6019
43	0.5112	0.4524	0.6018
44	0.5041	0.4524	0.6019
45	0.5105	0.4524	0.6019

(b) Hurricane Katrina in Louisiana and Mississippi

Period	SRB	Lower 95%	Upper 95%
57	0.5046	0.5043	0.5220
58	0.5145	0.5043	0.5220
59	0.5096	0.5042	0.5221
60	0.5169	0.5042	0.5221
61	0.5052	0.5041	0.5222
62	0.5142	0.5040	0.5222
63	0.5156	0.5040	0.5223
64	0.5181	0.5039	0.5224
65	0.5082	0.5039	0.5224
66	0.5110	0.5038	0.5225

(c) Virginia Tech Shooting in all states

Period	SRB	Lower 95%	Upper 95%
57	0.5110	0.4886	0.5437
58	0.5203	0.4886	0.5437
59	0.5026	0.4886	0.5437
60	0.5280	0.4886	0.5437
61	0.5324	0.4886	0.5437
62	0.5092	0.4886	0.5437
63	0.5315	0.4886	0.5437
64	0.5129	0.4886	0.5437
65	0.5139	0.4886	0.5437
66	0.5344	0.4886	0.5437

(d) Virginia Tech Shooting in adjacent states

Table C: Out-of-sample forecasts for the first 10 months after the intervention using SRB data grouped into 28-day periods and fitted with seasonal ARIMA models. Any period of which the observed SRB is outside of the 95% confidence level is marked by an asterisk (*).

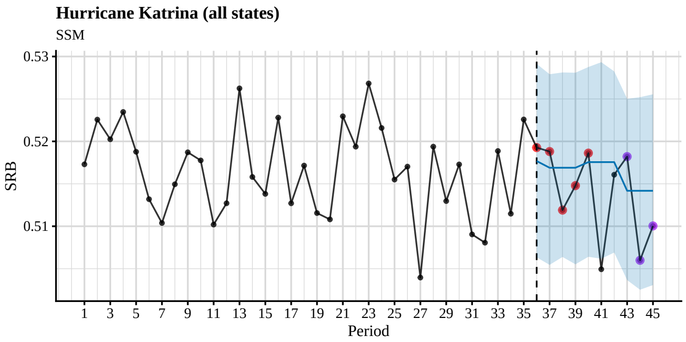

(a) Hurricane Katrina, all states

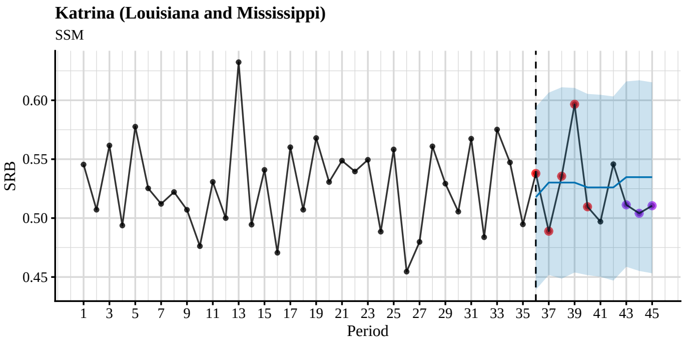

(b) Hurricane Katrina, Louisiana and Mississippi only

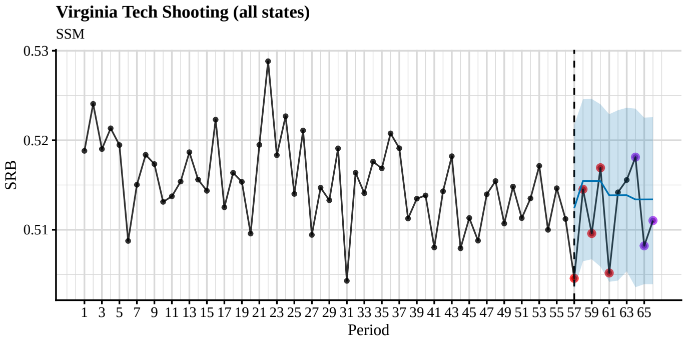

(c) Virginia Tech shooting, all states

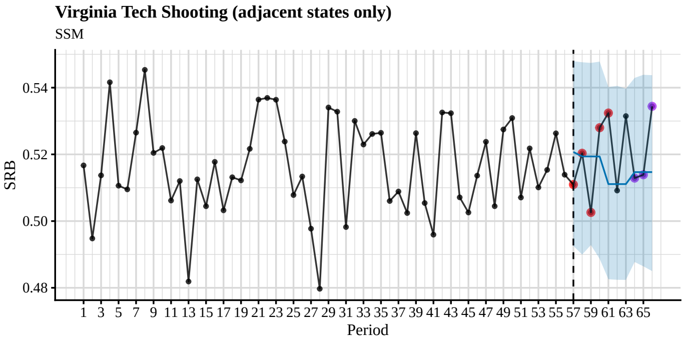

(d) Virginia Tech shooting, adjacent states only

Figure D: Time series plots and out-of-sample forecasts for SRB data grouped into 28-day periods and fitted with state-space models. The blue shade is the 95% confidence level. The observed SRBs for the first 5 months after the intervention are presented by red dots, whereas the observed SRBs for 7–9 months after the intervention are presented by purple dots. See also Table D.

Period	SRB	Lower 95%	Upper 95%
36	0.5193	0.5063	0.5291
37	0.5188	0.5054	0.5279
38	0.5119	0.5064	0.5281
39	0.5148	0.5055	0.5281
40	0.5186	0.5064	0.5288
41	0.5049	0.5062	0.5293
42	0.5161	0.5069	0.5282
43	0.5182	0.5037	0.5250
44	0.5060	0.5025	0.5252
45	0.5100	0.5031	0.5255

(a) Hurricane Katrina in all states

Period	SRB	Lower 95%	Upper 95%
36	0.5379	0.4392	0.5944
37	0.4889	0.4517	0.6065
38	0.5356	0.4486	0.6111
39	0.5966	0.4538	0.6105
40	0.5096	0.4516	0.6054
41	0.4970	0.4502	0.6047
42	0.5458	0.4470	0.6033
43	0.5112	0.4587	0.6160
44	0.5041	0.4551	0.6170
45	0.5105	0.4532	0.6153

(b) Hurricane Katrina in Louisiana and Mississippi

Period	SRB	Lower 95%	Upper 95%
57	0.5046	0.5034	0.5217
58	0.5145	0.5065	0.5246
59	0.5096	0.5067	0.5246
60	0.5169	0.5059	0.5240
61	0.5052	0.5042	0.5229
62	0.5142	0.5043	0.5234
63	0.5156	0.5054	0.5236
64	0.5181	0.5036	0.5235
65	0.5082	0.5039	0.5225
66	0.5110	0.5039	0.5226

(c) Virginia Tech Shooting in all states

Period	SRB	Lower 95%	Upper 95%
57	0.5110	0.4924	0.5487
58	0.5203	0.4910	0.5478
59	0.5026	0.4926	0.5469
60	0.5280	0.4896	0.5481
61	0.5324	0.4832	0.5382
62	0.5092	0.4815	0.5390
63	0.5315	0.4829	0.5399
64	0.5129	0.4894	0.5432
65	0.5139	0.4866	0.5450
66	0.5344	0.4846	0.5433

(d) Virginia Tech Shooting in adjacent states

Table D: Out-of-sample forecasts for the first 10 months after the intervention using SRB data grouped into 28-day periods and fitted with state-space models. Any period of which the observed SRB is outside of the 95% confidence level is marked by an asterisk (*).

IV.2 Weekly

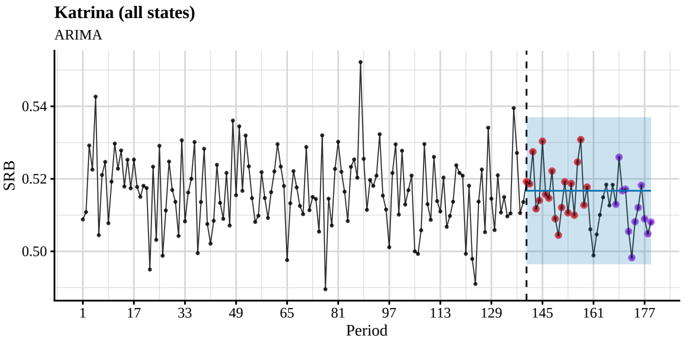

(a) Hurricane Katrina, all states

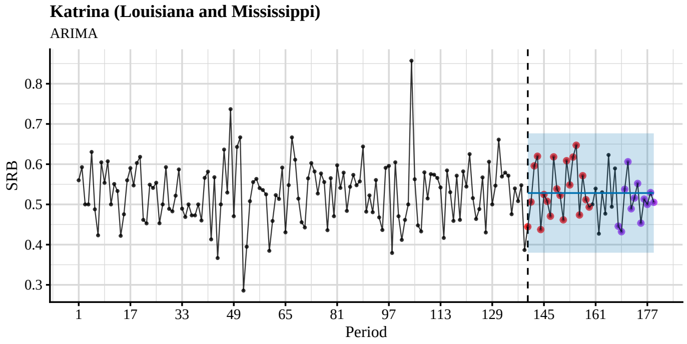

(b) Hurricane Katrina, Louisiana and Mississippi only

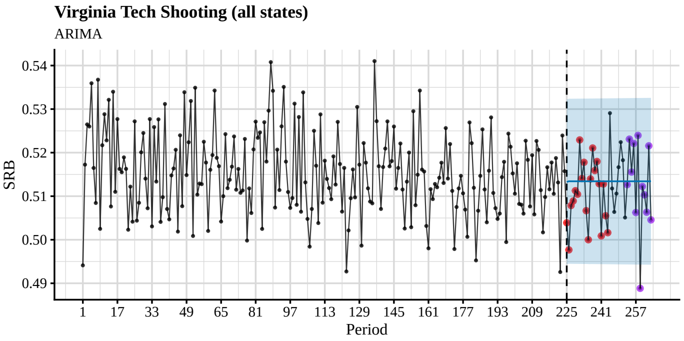

(c) Virginia Tech shooting, all states

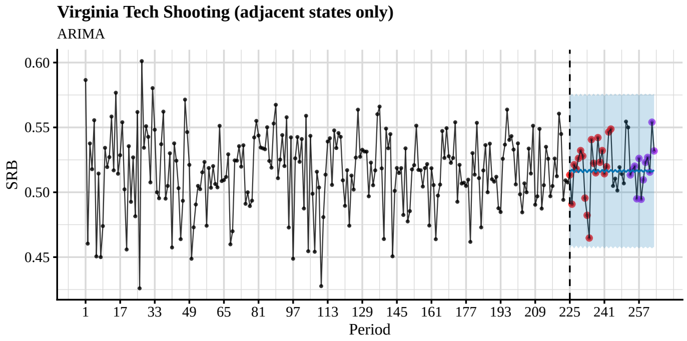

(d) Virginia Tech shooting, adjacent states only

Figure E: Time series plots and out-of-sample forecasts for SRB data grouped into 7-day periods and fitted with seasonal ARIMA models. The blue shade is the 95% confidence level. The observed SRBs for the first 5 months after the intervention are presented by red dots, whereas the observed SRBs for 7–9 months after the intervention are presented by purple dots. See also Table E.

Period	SRB	Lower 95%	Upper 95%
140	0.5192	0.4964	0.5370
141	0.5186	0.4964	0.5370
142	0.5275	0.4964	0.5370
143	0.5117	0.4964	0.5370
144	0.5140	0.4964	0.5370
145	0.5304	0.4964	0.5370
146	0.5157	0.4964	0.5370
147	0.5147	0.4964	0.5370
148	0.5222	0.4964	0.5370
149	0.5090	0.4964	0.5370
150	0.5045	0.4964	0.5370
151	0.5121	0.4964	0.5370
152	0.5192	0.4964	0.5370
153	0.5106	0.4964	0.5370
154	0.5187	0.4964	0.5370
155	0.5100	0.4964	0.5370
156	0.5246	0.4964	0.5370
157	0.5308	0.4964	0.5370
158	0.5128	0.4964	0.5370
159	0.5177	0.4964	0.5370
160	0.5061	0.4964	0.5370
161	0.4989	0.4964	0.5370
162	0.5047	0.4964	0.5370
163	0.5101	0.4964	0.5370
164	0.5149	0.4964	0.5370
165	0.5184	0.4964	0.5370
166	0.5127	0.4964	0.5370
167	0.5184	0.4964	0.5370
168	0.5130	0.4964	0.5370
169	0.5260	0.4964	0.5370
170	0.5167	0.4964	0.5370
171	0.5171	0.4964	0.5370
172	0.5055	0.4964	0.5370
173	0.4983	0.4964	0.5370
174	0.5082	0.4964	0.5370
175	0.5121	0.4964	0.5370
176	0.5182	0.4964	0.5370
177	0.5089	0.4964	0.5370
178	0.5049	0.4964	0.5370
179	0.5081	0.4964	0.5370

(a) Hurricane Katrina in all states

Period	SRB	Lower 95%	Upper 95%
140	0.4444	0.3799	0.6767
141	0.5060	0.3799	0.6767
142	0.5957	0.3799	0.6767
143	0.6197	0.3799	0.6767
144	0.4375	0.3799	0.6767
145	0.5246	0.3799	0.6767
146	0.5077	0.3799	0.6767
147	0.4706	0.3799	0.6767
148	0.6182	0.3799	0.6767
149	0.5385	0.3799	0.6767
150	0.5224	0.3799	0.6767
151	0.4615	0.3799	0.6767
152	0.6087	0.3799	0.6767
153	0.5484	0.3799	0.6767
154	0.6176	0.3799	0.6767
155	0.6471	0.3799	0.6767
156	0.4737	0.3799	0.6767
157	0.5714	0.3799	0.6767
158	0.5116	0.3799	0.6767
159	0.4933	0.3799	0.6767
160	0.5000	0.3799	0.6767
161	0.5393	0.3799	0.6767
162	0.4271	0.3799	0.6767
163	0.5301	0.3799	0.6767
164	0.4769	0.3799	0.6767
165	0.6230	0.3799	0.6767
166	0.4941	0.3799	0.6767
167	0.5895	0.3799	0.6767
168	0.4458	0.3799	0.6767
169	0.4321	0.3799	0.6767
170	0.5376	0.3799	0.6767
171	0.6061	0.3799	0.6767
172	0.4891	0.3799	0.6767
173	0.5158	0.3799	0.6767
174	0.5521	0.3799	0.6767
175	0.4535	0.3799	0.6767
176	0.5132	0.3799	0.6767
177	0.5000	0.3799	0.6767
178	0.5294	0.3799	0.6767
179	0.5052	0.3799	0.6767

(b) Hurricane Katrina in Louisiana and Mississippi

Period	SRB	Lower 95%	Upper 95%
225	0.5039	0.4943	0.5322
226	0.4977	0.4945	0.5324
227	0.5079	0.4945	0.5324
228	0.5089	0.4945	0.5324
229	0.5113	0.4945	0.5324
230	0.5104	0.4945	0.5324
231	0.5229	0.4944	0.5324
232	0.5141	0.4944	0.5324
233	0.5178	0.4944	0.5324
234	0.5067	0.4944	0.5324
235	0.5000	0.4944	0.5324
236	0.5140	0.4944	0.5324
237	0.5211	0.4944	0.5324
238	0.5159	0.4944	0.5324
239	0.5180	0.4944	0.5324
240	0.5129	0.4944	0.5325
241	0.5009	0.4944	0.5325
242	0.5128	0.4944	0.5325
243	0.5055	0.4944	0.5325
244	0.5016	0.4944	0.5325
245	0.5291	0.4944	0.5325
246	0.5118	0.4944	0.5325
247	0.5064	0.4944	0.5325
248	0.5106	0.4944	0.5325
249	0.5167	0.4943	0.5325
250	0.5224	0.4943	0.5325
251	0.5183	0.4943	0.5325
252	0.5051	0.4943	0.5325
253	0.5127	0.4943	0.5325
254	0.5231	0.4943	0.5325
255	0.5155	0.4943	0.5325
256	0.5221	0.4943	0.5325
257	0.5063	0.4943	0.5325
258	0.5240	0.4943	0.5325
259(*)	0.4888	0.4943	0.5326
260	0.5122	0.4943	0.5326
261	0.5103	0.4943	0.5326
262	0.5063	0.4943	0.5326
263	0.5216	0.4943	0.5326
264	0.5046	0.4943	0.5326

(c) Virginia Tech Shooting in all states

Period	SRB	Lower 95%	Upper 95%
225	0.5134	0.4567	0.5739
226	0.4908	0.4589	0.5761
227	0.5212	0.4567	0.5739
228	0.5179	0.4589	0.5761
229	0.5261	0.4567	0.5740
230	0.5322	0.4588	0.5761
231	0.5280	0.4567	0.5741
232	0.4955	0.4587	0.5761
233	0.4822	0.4567	0.5741
234	0.4647	0.4587	0.5761
235	0.5406	0.4567	0.5742
236	0.5223	0.4586	0.5761
237	0.5150	0.4567	0.5743
238	0.5421	0.4585	0.5761
239	0.5231	0.4567	0.5743
240	0.5322	0.4585	0.5761
241	0.5143	0.4567	0.5744
242	0.5197	0.4584	0.5761
243	0.5465	0.4567	0.5744
244	0.5487	0.4584	0.5761
245	0.5049	0.4567	0.5745
246	0.5105	0.4583	0.5761
247	0.5014	0.4567	0.5745
248	0.5193	0.4583	0.5761
249	0.5141	0.4567	0.5746
250	0.5068	0.4583	0.5761
251	0.5545	0.4567	0.5746
252	0.5500	0.4582	0.5761
253	0.5133	0.4567	0.5746
254	0.5172	0.4582	0.5761
255	0.5201	0.4567	0.5747
256	0.4949	0.4581	0.5761
257	0.5261	0.4567	0.5747
258	0.4946	0.4581	0.5761
259	0.5096	0.4567	0.5748
260	0.5231	0.4581	0.5761
261	0.5269	0.4568	0.5748
262	0.5155	0.4580	0.5761
263	0.5540	0.4568	0.5748
264	0.5318	0.4580	0.5761

(d) Virginia Tech Shooting in adjacent states

Table E: Out-of-sample forecasts for the first 10 months after the intervention using SRB data grouped into 7-day periods and fitted with seasonal ARIMA models. Any period of which the observed SRB is outside of the 95% confidence level is marked by an asterisk (*).

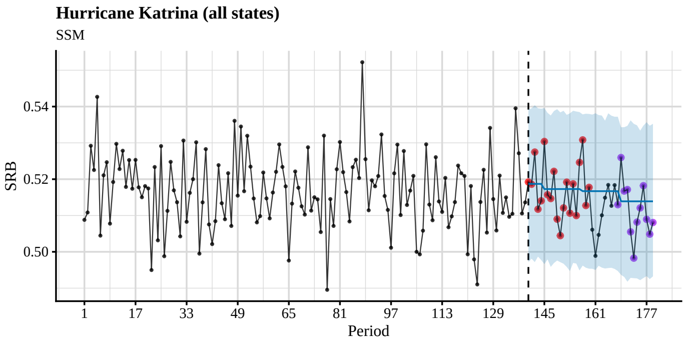

(a) Hurricane Katrina, all states

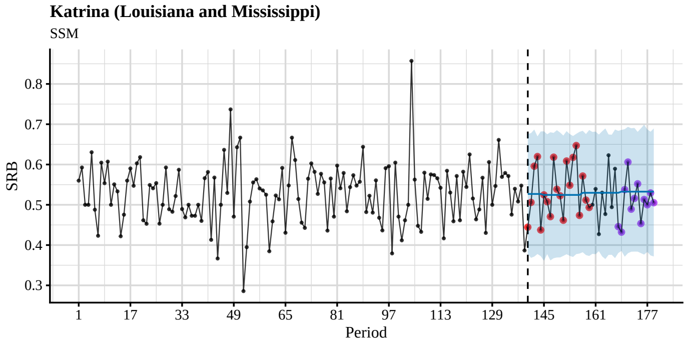

(b) Hurricane Katrina, Louisiana and Mississippi only

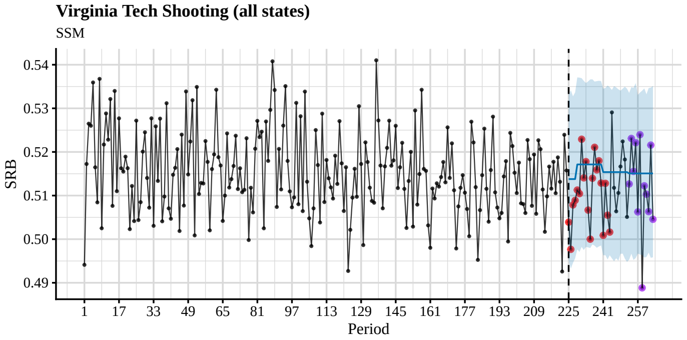

(c) Virginia Tech shooting, all states

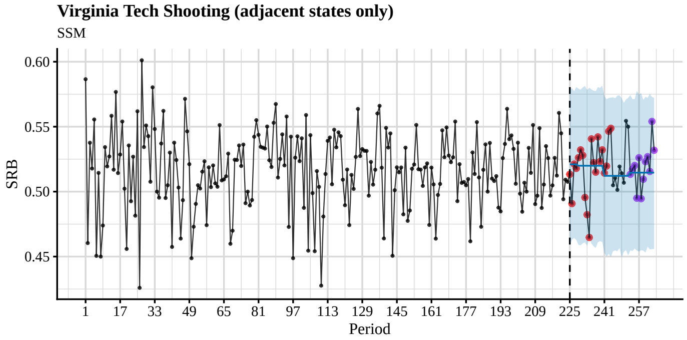

(d) Virginia Tech shooting, adjacent states only

Figure F: Time series plots and out-of-sample forecasts for SRB data grouped into 7-day periods and fitted with state-space models. The blue shade is the 95% confidence level. The observed SRBs for the first 5 months after the intervention are presented by red dots, whereas the observed SRBs for 7–9 months after the intervention are presented by purple dots. See also Table F.

Period	SRB	Lower 95%	Upper 95%
140	0.5192	0.4984	0.5388
141	0.5186	0.4971	0.5400
142	0.5275	0.4980	0.5384
143	0.5117	0.4974	0.5399
144	0.5140	0.4982	0.5394
145	0.5304	0.4951	0.5371
146	0.5157	0.4954	0.5392
147	0.5147	0.4963	0.5382
148	0.5222	0.4960	0.5387
149	0.5090	0.4949	0.5386
150	0.5045	0.4957	0.5378
151	0.5121	0.4970	0.5383
152	0.5192	0.4965	0.5390
153	0.5106	0.4961	0.5392
154	0.5187	0.4964	0.5390
155	0.5100	0.4967	0.5386
156	0.5246	0.4964	0.5369
157	0.5308	0.4958	0.5378
158	0.5128	0.4955	0.5366
159	0.5177	0.4952	0.5375
160	0.5061	0.4944	0.5392
161	0.4989	0.4950	0.5385
162	0.5047	0.4957	0.5377
163	0.5101	0.4953	0.5385
164	0.5149	0.4944	0.5382
165	0.5184	0.4958	0.5383
166	0.5127	0.4959	0.5368
167	0.5184	0.4959	0.5369
168	0.5130	0.4955	0.5370
169	0.5260	0.4922	0.5352
170	0.5167	0.4927	0.5339
171	0.5171	0.4926	0.5364
172	0.5055	0.4925	0.5340
173	0.4983	0.4924	0.5348
174	0.5082	0.4936	0.5351
175	0.5121	0.4934	0.5357
176	0.5182	0.4938	0.5347
177	0.5089	0.4917	0.5347
178	0.5049	0.4941	0.5351
179	0.5081	0.4928	0.5351

(a) Hurricane Katrina in all states

Period	SRB	Lower 95%	Upper 95%
140	0.4444	0.3793	0.6830
141	0.5060	0.3685	0.6803
142	0.5957	0.3726	0.6806
143	0.6197	0.3769	0.6859
144	0.4375	0.3680	0.6870
145	0.5246	0.3642	0.6749
146	0.5077	0.3789	0.6835
147	0.4706	0.3716	0.6780
148	0.6182	0.3691	0.6732
149	0.5385	0.3728	0.6870
150	0.5224	0.3742	0.6746
151	0.4615	0.3784	0.6823
152	0.6087	0.3665	0.6824
153	0.5484	0.3744	0.6809
154	0.6176	0.3691	0.6777
155	0.6471	0.3808	0.6810
156	0.4737	0.3655	0.6821
157	0.5714	0.3753	0.6799
158	0.5116	0.3833	0.6857
159	0.4933	0.3718	0.6812
160	0.5000	0.3801	0.6791
161	0.5393	0.3729	0.6883
162	0.4271	0.3790	0.6797
163	0.5301	0.3742	0.6746
164	0.4769	0.3739	0.6827
165	0.6230	0.3781	0.6797
166	0.4941	0.3730	0.6901
167	0.5895	0.3765	0.6865
168	0.4458	0.3765	0.6815
169	0.4321	0.3759	0.6864
170	0.5376	0.3806	0.6869
171	0.6061	0.3742	0.6965
172	0.4891	0.3844	0.6839
173	0.5158	0.3761	0.6805
174	0.5521	0.3795	0.6878
175	0.4535	0.3740	0.6792
176	0.5132	0.3797	0.6959
177	0.5000	0.3704	0.6787
178	0.5294	0.3742	0.6895
179	0.5052	0.3720	0.6802

(b) Hurricane Katrina in Louisiana and Mississippi

Period	SRB	Lower 95%	Upper 95%
225	0.5039	0.4926	0.5310
226	0.4977	0.4939	0.5313
227	0.5079	0.4941	0.5305
228	0.5089	0.4934	0.5306
229	0.5113	0.4954	0.5342
230	0.5104	0.4964	0.5354
231	0.5229	0.4972	0.5349
232	0.5141	0.4953	0.5337
233	0.5178	0.4954	0.5355
234	0.5067	0.4977	0.5344
235	0.5000	0.4966	0.5355
236	0.5140	0.4955	0.5351
237	0.5211	0.4967	0.5349
238	0.5159	0.4958	0.5350
239	0.5180	0.4970	0.5354
240	0.5129	0.4963	0.5352
241	0.5009	0.4943	0.5331
242	0.5128	0.4945	0.5323
243	0.5055	0.4938	0.5332
244	0.5016	0.4948	0.5322
245	0.5291	0.4940	0.5333
246	0.5118	0.4947	0.5342
247	0.5064	0.4946	0.5331
248	0.5106	0.4952	0.5328
249	0.5167	0.4944	0.5332
250	0.5224	0.4940	0.5335
251	0.5183	0.4948	0.5322
252	0.5051	0.4942	0.5333
253	0.5127	0.4948	0.5324
254	0.5231	0.4936	0.5325
255	0.5155	0.4936	0.5325
256	0.5221	0.4947	0.5335
257	0.5063	0.4938	0.5341
258	0.5240	0.4947	0.5327
259(*)	0.4888	0.4944	0.5327
260	0.5122	0.4946	0.5337
261	0.5103	0.4944	0.5326
262	0.5063	0.4936	0.5337
263	0.5216	0.4938	0.5326
264	0.5046	0.4938	0.5341

(c) Virginia Tech Shooting in all states

Period	SRB	Lower 95%	Upper 95%
225	0.5134	0.4612	0.5784
226	0.4908	0.4613	0.5839
227	0.5212	0.4622	0.5824
228	0.5179	0.4640	0.5814
229	0.5261	0.4644	0.5794
230	0.5322	0.4627	0.5820
231	0.5280	0.4604	0.5799
232	0.4955	0.4590	0.5822
233	0.4822	0.4609	0.5793
234	0.4647	0.4554	0.5789
235	0.5406	0.4622	0.5778
236	0.5223	0.4625	0.5801
237	0.5150	0.4609	0.5819
238	0.5421	0.4593	0.5778
239	0.5231	0.4603	0.5750
240	0.5322	0.4610	0.5764
241	0.5143	0.4519	0.5705
242	0.5197	0.4484	0.5681
243	0.5465	0.4514	0.5725
244	0.5487	0.4530	0.5713
245	0.5049	0.4547	0.5747
246	0.5105	0.4553	0.5755
247	0.5014	0.4525	0.5729
248	0.5193	0.4528	0.5704
249	0.5141	0.4513	0.5735
250	0.5068	0.4526	0.5735
251	0.5545	0.4548	0.5722
252	0.5500	0.4525	0.5697
253	0.5133	0.4515	0.5723
254	0.5172	0.4514	0.5741
255	0.5201	0.4574	0.5718
256	0.4949	0.4575	0.5749
257	0.5261	0.4548	0.5744
258	0.4946	0.4553	0.5765
259	0.5096	0.4553	0.5750
260	0.5231	0.4550	0.5745
261	0.5269	0.4571	0.5737
262	0.5155	0.4568	0.5762
263	0.5540	0.4540	0.5755
264	0.5318	0.4576	0.5720

(d) Virginia Tech Shooting in adjacent states

Table F: Out-of-sample forecasts for the first 10 months after the intervention using SRB data grouped into 7-day periods and fitted with state-space models. Any period of which the observed SRB is outside of the 95% confidence level is marked by an asterisk (*).

V Swedish SRB and Meteorological Observations

Using the data downloaded from the World Bank (https://climateknowledgeportal.worldbank.org/download-data), we performed a Pearson's correlation test and a Granger causality test. The p-values for the null hypotheses of the nonexistence of correlation (Student's t-test) and Granger causality (F-test) are listed in Table G. We could not establish associations between the SRB and either of the meteorological observations between years 1991 and 2013.

	Temperature	Precipitation
t-test	0.156	0.765
F-test	0.269	0.228

Table G: p-values for t- and F-tests on the correlation between Sweden's SRB and temperature and precipitation in Sweden

In addition, we performed logistic regression using the following:

Factor	ΔIC	SE
At risk of poverty	0.9934	3.5615
SO_2	0.8399	3.6321
NO_2	-0.5075	3.2862
Proportion foreign nationals	-1.8912	2.2786
Black smoke	-2.0551	2.7690
P80/P20	-2.2833	2.2427
Car density	-2.6865	2.4586
Population density	-2.7654	2.1618
Gini	-2.7838	2.2333
Mean income	-3.4858	2.1006
PAH	-3.7904	2.0331
VOC	-4.3064	1.8581
PM_{10}	-4.4771	1.7049
$PM_{2.5}$	-4.9524	1.4270
Median income	-5.1749	1.9721

Table H: Differences in information criteria (ΔIC) and their standard errors (SE) of individual factors at the kommun (municipality) level, with random effect at the län (county) level

Table I: Differences in information criteria (ΔIC) and their standard errors (SE) of individual factors at the län (county) level

Factor	ΔIC	SE
diseases of the respiratory system men	3.3012	2.5949
good health men and women	1.4435	2.4498
diseases of the circulatory system women	1.1142	3.2312
serious motor disabilities women	0.9167	3.3112
smoke and or take snuff daily men	0.8522	2.1592
unmet need for medical care men	0.7556	2.4153
motor disabilities men	0.6604	3.1667
high blood pressure women	0.2627	3.0594
smoke and or take snuff daily women	0.2378	3.1951
impaired hearing men	0.0979	2.7779
diabetes men	0.0969	3.1908
diseases of the circulatory system men	0.0838	2.7351
diseases of the respiratory system men and women	-0.1382	2.9142
serious pain total men and women	-0.1481	2.3953
serious motor disabilities men and women	-0.1740	3.0316
serious problems of anxiety worry fear men and women	-0.2266	3.5719
unmet need for medical care women	-0.2308	2.5992
poor health women	-0.3233	2.2780
smoke daily women	-0.3289	3.2831
impaired hearing women	-0.5162	2.7654
problems of anxiety worry fear men	-0.5694	2.2561
impaired vision men and women	-0.7338	2.4952
obese BMI 30 or more men	-1.0034	3.1003
serious motor disabilities men	-1.0759	2.2428
serious pain in shoulders neck women	-1.1545	2.2927
impaired vision women	-1.1893	2.4814
serious pain in hands elbows or knees men	-1.2649	3.0558
smoke daily men	-1.5168	2.7761
diseases of the musculoskeletal system and connective tissue men	-1.5226	2.4445
diseases of the skin men and women	-1.6969	3.6015
diseases of the musculoskeletal system and connective tissue men and women	-1.7217	2.6642
diabetes women	-1.8868	2.4746
severe problems from a long term illness women	-2.1122	2.7674
serious pain in shoulders neck men	-2.1671	2.8550
trouble sleeping men and women	-2.1719	2.1819
endocrine diseases men and women	-2.1860	2.4510
trouble sleeping women	-2.1972	3.5518
endocrine diseases women	-2.3205	2.5285
dentist appointments during a 12 month period men and women	-2.3418	2.8350
serious pain total men	-2.3436	4.0538
problems of anxiety worry fear men and women	-2.4328	2.5539
poor health men	-2.4561	2.3493
serious pain in back or hips men and women	-2.6307	3.2649

Continued on next page

Table I: Differences in information criteria (ΔIC) and their standard errors (SE) of individual factors at the län (county) level

Factor	ΔIC	SE
diseases of the circulatory system men and women	-2.7013	2.3490
high blood pressure men and women	-2.7124	2.1525
overweight or obese BMI 25 or more men and women	-2.7407	2.4722
serious pain total women	-2.7984	2.2393
diseases of the musculoskeletal system and connective tissue women	-2.8772	2.9321
diseases of the skin men	-3.0005	2.7985
dentist appointments during a 12 month period men	-3.0185	2.9935
unmet need for medical care men and women	-3.0341	1.7253
serious pain in hands, elbows, or knees women	-3.1847	3.1895
diabetes men and women	-3.2320	2.2913
serious problems of anxiety, worry, fear men	-3.2685	2.7144
serious pain in shoulders, neck men and women	-3.2852	2.7533
doctor appointments during a three month period men	-3.3952	1.9437
heart disease men and women	-3.4409	2.5030
overweight or obese BMI 25 or more men	-3.4821	2.5026
motor disabilities women	-3.5407	3.4502
obese BMI 30 or more men and women	-3.6252	2.4101
doctor appointments during a three month period men and women	-3.6527	1.6407
endocrine diseases men	-3.7796	2.4763
overweight or obese BMI 25 0 or more women	-3.9447	2.8735
severe problems from a long term illness men and women	-3.9898	2.9988
good health women	-4.0257	2.8712
heart disease men	-4.1542	2.1345
poor health men and women	-4.3023	3.0782
take snuff daily men and women	-4.3065	3.0176
problems of anxiety worry fear women	-4.3155	2.2453
serious pain in hands elbows or knees men and women	-4.3937	2.3697
impaired hearing men and women	-4.5371	2.0341
diseases of the digestive system men and women	-4.6030	2.6724
serious pain in back or hips women	-4.6210	2.2235
severe problems from a long term illness men	-4.7071	2.3077
dentist appointments during a 12 month period women	-4.7839	2.6277
take snuff daily men	-4.9910	2.1400
impaired vision men	-5.1140	2.2114
unmet need for dental care men	-5.6746	2.5992
diseases of the respiratory system women	-5.7384	2.5305
high blood pressure men	-5.7527	3.8451
serious pain in back or hips men	-5.8251	3.5053
diseases of the digestive system men	-5.9368	2.6694
unmet need for dental care men and women	-6.1628	1.9368
heart disease women	-6.2097	1.9162
take snuff daily women	-6.3476	2.0574
obese BMI 30 0 or more women	-6.4418	2.0136
motor disabilities men and women	-6.7537	1.9335

Continued on next page

Table I: Differences in information criteria (ΔIC) and their standard errors (SE) of individual factors at the län (county) level

Factor	ΔIC	SE
smoke and or take snuff daily men and women	-6.7639	2.4234
trouble sleeping men	-6.7890	3.4973
smoke daily men and women	-6.8021	4.0868
diseases of the nervous system and the sensory organs men	-6.8766	1.8000
diseases of the skin women	-7.0391	2.9732
diseases of the nervous system and the sensory organs men and women	-7.0903	2.1524
diseases of the digestive system women	-7.0938	2.0396
doctor appointments during a three month period women	-7.1649	4.1678
diseases of the nervous system and the sensory organs women	-7.1994	2.4814
good health men	-7.4083	1.7240
unmet need for dental care women	-8.1221	3.4038
serious problems of anxiety worry fear women	-8.7865	1.7444

VI Contingency Table Analysis

Table J is the full contingency table for testing the association between physical injury, infections, and neuropsychiatric disorders (stratified by before/during diagnosis) and SRB.

Phys_1y	Infection_1y	Neuropsych_1y	Phys_older	Infection_older	Neuropsych_older	F	M	Total
0	0	0	0	0	0	693079	733474	1426553
0	0	0	0	0	1	16835	17539	34374
0	0	0	0	1	0	65169	68766	133935
0	0	0	0	1	1	15297	16215	31512
0	0	0	1	0	0	5700	5945	11645
0	0	0	1	0	1	1265	1420	2685
0	0	0	1	1	0	5031	5250	10281
0	0	0	1	1	1	2424	2565	4989
0	0	1	0	0	0	30176	31833	62009
0	0	1	0	0	1	10905	11480	22385
0	0	1	0	1	0	5456	5688	11144
0	0	1	0	1	1	8629	9092	17721
0	0	1	1	0	0	464	537	1001
0	0	1	1	0	1	808	857	1665
0	0	1	1	1	0	510	477	987
0	0	1	1	1	1	1488	1554	3042
0	1	0	0	0	0	65857	69326	135183
0	1	0	0	0	1	3926	4229	8155
0	1	0	0	1	0	24502	25721	50223
0	1	0	0	1	1	6555	6952	13507
0	1	0	1	0	0	1230	1241	2471
0	1	0	1	0	1	352	385	737
0	1	0	1	1	0	1962	1993	3955
0	1	0	1	1	1	1077	1185	2262
0	1	1	0	0	0	9609	10417	20026
0	1	1	0	0	1	3666	3960	7626
0	1	1	0	1	0	3301	3422	6723
0	1	1	0	1	1	5243	5653	10896
0	1	1	1	0	0	174	173	347
0	1	1	1	0	1	312	316	628
0	1	1	1	1	0	271	313	584
0	1	1	1	1	1	977	1006	1983
1	0	0	0	0	0	5894	6180	12074
1	0	0	0	0	1	434	420	854
1	0	0	0	1	0	1484	1565	3049
1	0	0	0	1	1	426	469	895
1	0	0	1	0	0	245	245	490
1	0	0	1	0	1	69	70	139
1	0	0	1	1	0	227	221	448
1	0	0	1	1	1	117	138	255
1	0	1	0	0	0	1329	1454	2783
1	0	1	0	0	1	527	550	1077
1	0	1	0	1	0	258	286	544
1	0	1	0	1	1	436	482	918
1	0	1	1	0	0	28	50	78
1	0	1	1	0	1	195	203	398
1	0	1	1	1	0	28	43	71
1	0	1	1	1	1	235	256	491
1	1	0	0	0	0	2122	2204	4326
1	1	0	0	0	1	181	182	363
1	1	0	0	1	0	929	903	1832
1	1	0	0	1	1	288	305	593
1	1	0	1	0	0	69	79	148
1	1	0	1	0	1	27	31	58
1	1	0	1	1	0	143	134	277
1	1	0	1	1	1	81	87	168
1	1	1	0	0	0	650	713	1363
1	1	1	0	0	1	249	263	512
1	1	1	0	1	0	214	226	440
1	1	1	0	1	1	408	427	835
1	1	1	1	0	0	22	15	37
1	1	1	1	0	1	66	84	150
1	1	1	1	1	0	35	46	81
1	1	1	1	1	1	204	203	407
Total						1009870	1067518	2077388

Table J: Contingency table of maternal diagnosis history versus the sex of livebirths

VII Dictionary of factors and their definitions

Table K: List of variable names used in the main text and their corresponding definitions and units (if applicable)

Variable Name	Variable Definition	Units
A_PM10_mean_ln	particulate matter under ten micrometers in aerodynamic diameter (PM10)	ln-μg/m3
A_PM25_mean	particulate matter under 2.5 micrometers in aerodynamic diameter (PM2.5)	ln-μg/m3
A_NO2_mean_ln	nitrogen dioxide (NO2)	ln-ppb
A_SO2_mean_ln	sulfur dioxide (SO2)	ln-ppb
A_O3_mean_ln	ozone (O3)	ln-ppm
A_CO_mean_ln	carbon monoxide (CO)	ln-ppm
A_TeCA_ln	1,1,2,2-tetrachloroethane	ln-tons
A_112TCA_ln	1,1,2-trichloroethane	ln-tons
A_DBCP_ln	1,2-dibromo-3-chloropropane	ln-tons
A_TDI_ln	2,4-toluene diisocyanate	ln-tons
A_2Clacephen_ln	2-chloroacetophenone	ln-tons
A_2NP_ln	2-nitropropane	ln-tons
A_PNP_ln	4-nitrophenol	ln-tons
A_CH3CN_ln	acetonitrile	ln-tons
A_Acetophenone_ln	acetophenone	ln-tons
A_Acrolein_ln	acrolein	ln-tons
A_Acrylic_acid_ln	acrylic acid	ln-tons
A_C3H3N_ln	acrylonitrile	ln-tons
A_Sb_ln	antimony compounds	ln-tons
A_Benzidine_ln	benzidine	ln-tons
A_Benzyl_Cl_ln	benzyl chloride	ln-tons
A_Be_ln	beryllium compounds	ln-tons
A_biphenyl_ln	biphenyl	ln-tons
A_DEHP_ln	bis-2-ethylhexyl phthalate	ln-tons
A_Bromoform_ln	bromoform	ln-tons
A_Cd_ln	cadmium compounds	ln-tons
A_CS2_ln	carbon disulfide	ln-tons
A_CCl4	carbon tetrachloride	tons
A_CS_ln	carbon sulfide	ln-tons
A_Cl_ln	chlorine	ln-tons
A_C6H5Cl_ln	chlorobenzene	ln-tons
A_chloroform_ln	chloroform	ln-tons
A_Chloroprene_ln	chloroprene	ln-tons
A_Cr_ln	chromium compounds	ln-tons
A_Cresol_ln	cresol/cresylic acid	ln-tons
A_Cumene_ln	cumene	ln-tons
A_CN_ln	cyanide compounds	ln-tons
A_DBP_ln	dibutylphthalate	ln-tons
A_Diesel_ln	diesel engine emissions	ln-tons
A_DMF_ln	dimethyl formamide	ln-tons
A_Me2_phthalate_ln	dimethyl phthalates	ln-tons
A_Me2SO4_ln	dimethyl sulfate	ln-tons
A_ECH_ln	epichlorohydrin	ln-tons
A_Etacrylate_ln	ethyl acrylate	ln-tons
A_EtCl_ln	ethyl chloride	ln-tons
A_EDB_ln	ethylene dibromide	ln-tons
A_EDC_ln	ethylene dichloride	ln-tons
A_EGLY_ln	ethylene glycol	ln-tons
A_EOx_ln	ethylene oxide	ln-tons
A_EdCl2_ln	ethylidene dichloride	ln-tons
A_Glycol_ethers_ln	glycol ethers	ln-tons
A_HCB_ln	hexachlorobenzene	ln-tons
A_HCBD_ln	hexachlorobutadiene	ln-tons
A_HCCPD_ln	hexachlorocyclopentadiene	ln-tons
A_Hexane_ln	hexane	ln-tons
A_N2H2_ln	hydrazine	ln-tons
A_HCl_ln	hydrochloric acid	ln-tons
A_Isophorone_ln	isophorone	ln-tons
A_Pb_ln	lead compounds	ln-tons
A_Mn_ln	manganese compounds	ln-tons
A_Hg_ln	mercury compounds	ln-tons
A_MeOH_ln	methanol	ln-tons
A_MIBK_ln	methyl isobutyl ketone	ln-tons
A_MMA_ln	methyl methacrylate	ln-tons
A_MeCl_ln	methyl chloride	ln-tons
A_Mehydrazine_ln	methylhydrazine	ln-tons
A_MTBE_ln	MTBE	ln-tons
A_nitrobenzene_ln	nitrobenzene	ln-tons
A_DMA_ln	N,N-dimethylaniline	ln-tons
A_otoluidine_ln	o-toluidine	ln-tons
A_PAHPOM_ln	PAH/POM	ln-tons
A_PCP_ln	pentachlorophenol	ln-tons
A_PH3_ln	phosphine	ln-tons
A_P_ln	phosphorus	ln-tons
A_PCBs_ln	polychlorinated biphenyls	ln-tons
A_ProCl2_ln	propylene dichloride	ln-tons
A_ProO_ln	propylene oxide	ln-tons
A_Quinoline_ln	quinoline	ln-tons
A_Se_ln	selenium compounds	ln-tons
A_Styrene_ln	styrene	ln-tons
A_Cl4C2_ln	tetrachloroethylene	ln-tons
A_Toluene_ln	toluene	ln-tons
A_C2HCl3_ln	trichloroethylene	ln-tons
A_Et3N_ln	triethylamine	ln-tons
A_VyAc_ln	vinyl acetate	ln-tons

Continued on next page

Table K: List of variable names used in the main text and their corresponding definitions and units (if applicable)

Variable Name	Variable Definition	Units
A_VyCl_ln	vinyl chloride	ln-tons
A_11DCE_ln	vinylidene chloride	ln-tons
D303_Percent	% of stream length impaired in county	percent
SEWAGENPDESperKM	sewage permits per 1000 km of stream in county	permits/1000km
INDNPDESperKM	industrial permits per 1000 km of stream in county	permits/1000km
STORMNPDESperKM	stormwater permits per 1000 km of stream in county	permits/1000km
numDays_Close_Activity_tot	# of days closed per event in county 2000-2005	days
numDays_Cont_Activity_tot	# of days per contamination advisory event in county 2000-2005	days
numDays_Rain_Activity_tot	# of days per rain advisory event in county 2000-2005	days
Per_TotPopSS_ave	percent of population on self supply, average 2000&2005	percent
Per_PSWithSW_ave	percent of public supply population which is on surface water, average 2000&2005	percent
Ca_ln_ave	calcium (Ca) precipitation weighted mean	ln mg/L
Mg_ln_ave	magnesium (Mg) precipitation weighted mean	ln mg/L
K_ln_ave	potassium (K) precipitation weighted mean	ln mg/L
Na_ln_ave	sodium (Na) precipitation weighted mean	ln mg/L
NH4_mean_ave	ammonium (NH4) precipitation weighted mean	mg/L
NO3_mean_ave	nitrate (NO3) precipitation weighted mean	mg/L
Cl_ln_ave	chloride (Cl) precipitation weighted mean	ln mg/L
SO4_mean_ave	sulfate (SO4) precipitation weighted mean	mg/L
Hg_ln_ave	total mercury (Hg) deposition	ln mg/L
AvgOfD3_ave	% of county drought – extreme (3-D4)	percent
W_As_ln	arsenic	ln mg/L
W_Ba_ln	barium	ln mg/L
W_Cd_ln	cadmium	ln mg/L
W_Cr_ln	chromium	ln mg/L
W_CN_ln	cyanide	ln mg/L
W_FL_ln	fluoride	ln mg/L
W_HG_ln	mercury (inorganic)	ln mg/L
W_NO3_ln	nitrate	ln mg/L
W_NO2_ln	nitrite	ln mg/L
W_SE_ln	selenium	ln mg/L
W_Sb_ln	antimony	ln mg/L
W_Be_ln	beryllium	ln mg/L
W_Tl_ln	thallium	ln mg/L
W_Endrin_ln	endrin	ln mg/L
W_Lindane_ln	lindane	ln mg/L
W_methoxychlor_ln	methoxychlor	$\ln \mu g/L$
W_Toxaphene_ln	toxaphene	$\ln \mu g/L$
W_Dalapon_ln	dalapon	$\ln \mu g/L$
W_DEHA_ln	di(2-ethylhexyl)adipate (DEHA)	$\ln \mu g/L$
W_Oxamyl_ln	oxamyl (Vydate)	$\ln \mu g/L$
W_Simazine_ln	simazine	$\ln \mu g/L$
W_DEHP_ln	Di(2-ethylhexyl) phthalate (DEHP)	$\ln \mu g/L$
W_Picloram_ln	picloram	$\ln \mu g/L$
W_Dinoseb_ln	dinoseb	$\ln \mu g/L$
W_HCCPD_ln	hexachlorocyclopentadiene	$\ln \mu g/L$
W_Carbofuran_ln	carbofuran	$\ln \mu g/L$
W_atrazine_ln	atrazine	$\ln \mu g/L$
W_Alachlor_ln	alachlor	$\ln \mu g/L$
W_Heptachlor_ln	heptachlor	$\ln \mu g/L$
W_Heptachlor_epox_ln	heptachlor epoxide	$\ln \mu g/L$
W_24D_ln	2,4-D (2,4-Dichlorophenoxyacetic acid)	$\ln \mu g/L$
W_HCB_ln	hexachlorobenzene	$\ln \mu g/L$
W_BenzoAP_ln	benzo[a]pyrene	$\ln \mu g/L$
W_PCP_ln	pentachlorophenol	$\ln \mu g/L$
W_124TCIB_ln	1,2,4-trichlorobenzene	$\ln \mu g/L$
W_PCB_ln	polychlorinated biphenyls (PCBs)	$\ln \mu g/L$
W_DBCP_ln	1,2-dibromo-3-chloropropane (DBCP)	$\ln \mu g/L$
W_EBD_ln	ethylene dibromide (EDB)	$\ln \mu g/L$
W_xylenes_ln	xylenes	$\ln \mu g/L$
W_Chlordane_ln	chlordane	$\ln \mu g/L$
W_DCM_ln	dichloromethane (methylene chloride)	$\ln \mu g/L$
W_ODCB_ln	1,2-dichlorobenzene (o-dichlorobenzene)	$\ln \mu g/L$
W_PDCB_ln	1,4-dichlorobenzene (p-dichlorobenzene)	$\ln \mu g/L$
W_VCM_ln	vinyl chloride	$\ln \mu g/L$
W_11DCE_ln	1,1-dichloroethylene	$\ln \mu g/L$
W_t12DCE_ln	trans-1,2-Dichloroethylene	$\ln \mu g/L$
W_EDC_ln	1,2-dichloroethane (Ethylene Dichloride)	$\ln \mu g/L$
W_111trichlorane_ln	1,1,1-trichloroethane	$\ln \mu g/L$
W_CCl4_ln	carbon tetrachloride	$\ln \mu g/L$
W_PDC_ln	1,2-dichloropropane	$\ln \mu g/L$
W_Trichlorene_ln	trichloroethylene	$\ln \mu g/L$
W_112TCA_ln	1,1,2-trichloroethane	$\ln \mu g/L$
W_C2Cl4_ln	tetrachloroethylene	$\ln \mu g/L$
W_benzene_ln	benzene	$\ln \mu g/L$
W_Cl1benz_ln	monochlorobenzene (chlorobenzene)	$\ln \mu g/L$
W_Toluene_ln	toluene	$\ln \mu g/L$
W_ethylbenz_ln	ethylbenzene	$\ln \mu g/L$
W_styrene_ln	styrene	$\ln \mu g/L$
W_DCE_ln	alpha particles	$\ln \mu g/L$
W_alpha	cis-1,2-dichloroethylene	PCl/L
W_SILVEX_ln	silvex	$\ln \mu g/L$
pct_harvest_acres	harvested acreage	percent
pct_irrigated_acres_ln	irrigated acreage	ln-percent
farms_per_acre_ln	farms per acre	ln-(number farms/total acres)
pct_manure_acres_ln	manure applied	ln-percent
pct_nematode_acres_ln	chemicals used to control nematodes	ln-percent
pct_disease_acres_ln	chemicals used to control disease	ln-percent
pct_defoliate_acres_ln	chemicals used to defoliate/control growth/thin fruit	ln-percent
pct_au_ln	animal units	ln-percent

Continued on next page

Table K: List of variable names used in the main text and their corresponding definitions and units (if applicable)

Variable Name	Variable Definition	Units
herbicides_ln	herbicides	ln-pounds
fungicides_ln	fungicides	ln-pounds
insecticides_ln	insecticides	ln-pounds
mean_as_ln	arsenic	ln-ppm
mean_se_ln	selenium	ln-ppm
mean_hg_ln	mercury	ln-ppm
mean_pb_ln	lead	ln-ppm
mean_zn_ln	zinc	ln-ppm
mean_cu_ln	copper	ln-ppm
mean_na__pct_ln	sodium	ln-weighted percent
mean_mg_pct_ln	magnesium	ln-weighted percent
mean_ti_pct_ln	titanium	ln-weighted percent
mean_ca_pct_ln	calcium	ln-weighted percent
mean_fe_pct_ln	iron	ln-weighted percent
mean_al_pct	aluminum	weighted percent
mean_p_pct	phosphorus	weighted percent
facilities_rate_log	facilities per county pop	ln-rate
radon_zone	radon zone	radon category
HWYPROP	proportion of roads that are highway	miles highways / miles total roads
RYPROP	proportion of roads that are primary streets	miles primary streets / miles total roads
fatal_rate_log	traffic fatality rate	ln-rate
pct_pub_transport_log	percent of population using public transport	ln-percent
rate_al_pn_gm_env_log	vice-related businesses	ln-rate
rate_ent_env_log	entertainment-related businesses	ln-rate
rate_ed_env_log	education-related businesses	ln-rate
rate_food_env_neg	negative food related businesses	rate
rate_food_env_pos_log	positive food related businesses	ln-rate
rate_hc_env_log	health care-related businesses	ln-rate
rate_rec_env_log	recreation-related businesses	ln-rate
rate_trans_env_log	transportation-related businesses	ln-rate
rate_civic_env_log	civic-related businesses	ln-rate
to_unit_rate_log	total subsidized housing units	ln-rate
pct_rent_occ	percent renter occupied	count county occupied rental units / total county units
pct_vac_units	percent vacant units	count county vacant units / total housing units
med_hh_value	median household value	dollars
med_hh_inc	median household income	dollars
pct_pers_lt_pov	percent persons less than poverty level	count county persons below poverty / county population
pct_no_eng	percent no English	count county non-English speaking / county population
pct_hs_more	percent earning greater than high school education	count county more than high school / county population
pct_unemp	percent unemployed	count county unemployed / county population
work_out_co	percent work outside county	count county work outside county / county population
med_rooms	median number rooms per house	sum county count of rooms / county housing units
pct_mt_10units_log	percent of housing with more than 10 units	ln-percent
violent_rate_log	mean number of violent crimes per capita	ln-rate
fips	FIPS code to state and county level	N/A
county_name	name of county	N/A
state	state	N/A
cat_rucc	rural-urban continuum code category	N/A
air_EQI_22July2013	non-stratified air domain index	N/A
water_EQI_22July2013	non-stratified water domain index	N/A
land_EQI_22July2013	non-stratified land domain index	N/A
sociod_EQI_22July2013	non-stratified sociodemographic domain index	N/A
built_EQI_22July2013	non-stratified built environment domain index	N/A
EQI_22July2013	non-stratified environmental quality index	N/A
RUCC1_air_EQI_22July2013	metropolitian-urbanized strata air domain index	N/A
RUCC1_water_EQI_22July2013	metropolitian-urbanized strata water domain index	N/A
RUCC1_land_EQI_22July2013	metropolitian-urbanized strata land domain index	N/A
RUCC1_sociod_EQI_22July2013	metropolitian-urbanized strata sociodemographic domain index	N/A
RUCC1_built_EQI_22July2013	metropolitian-urbanized strata built environment domain index	N/A
RUCC1_EQI_22July2013	metropolitian-urbanized strata environmental quality index	N/A
RUCC2_air_EQI_22July2013	non-metropolitian-urbanized strata air domain index	N/A
RUCC2_water_EQI_22July2013	non-metropolitian-urbanized strata water domain index	N/A
RUCC2_land_EQI_22July2013	non-metropolitian-urbanized strata land domain index	N/A
RUCC2_sociod_EQI_22July2013	non-metropolitian-urbanized strata sociodemographic domain index	N/A
RUCC2_built_EQI_22July2013	non-metropolitian-urbanized strata built environment domain index	N/A
RUCC2_EQI_22July2013	non-metropolitian-urbanized strata environmental quality index	N/A
RUCC3_air_EQI_22July2013	less-urbanized strata air domain index	N/A
RUCC3_water_EQI_22July2013	less-urbanized strata water domain index	N/A
RUCC3_land_EQI_22July2013	less-urbanized strata land domain index	N/A
RUCC3_sociod_EQI_22July2013	less-urbanized strata sociodemographic domain index	N/A
RUCC3_built_EQI_22July2013	less-urbanized strata built environment domain index	N/A
RUCC3_EQI_22July2013	less-urbanized strata environmental quality index	N/A
RUCC4_air_EQI_22July2013	rural strata air domain index	N/A
RUCC4_water_EQI_22July2013	rural strata water domain index	N/A
RUCC4_land_EQI_22July2013	rural strata land domain index	N/A
RUCC4_sociod_EQI_22July2013	rural strata sociodemographic domain index	N/A
RUCC4_built_EQI_22July2013	rural strata built environment domain index	N/A
RUCC4_EQI_22July2013	rural strata environmental quality index	N/A
