# Supplementary material for: Observable variations in human sex ratio at birth
Source: PLoS Comput Biol. 2021 Dec 2;17(12):e1009586. doi: 10.1371/journal.pcbi.1009586 (PMC8638995; doi:10.1371/journal.pcbi.1009586)
Supplement: Attachment — Submitted filename: SRB_PLoS_comp_bio_rebuttal.pdf [file pcbi.1009586.s002.pdf]

Responses to Reviewers Version 1

Editor's comments

Thank you very much for submitting your manuscript "Observable Variations in Human Sex Ratio at Birth" for consideration at PLOS Computational Biology. As with all papers reviewed by the journal, your manuscript was reviewed by members of the editorial board and by several independent reviewers. The reviewers appreciated the attention to an important topic. Based on the reviews, we are likely to accept this manuscript for publication, providing that you modify the manuscript according to the review recommendations.

I apologise for the long wait for reviews, with which there were some difficulties.

Both reviewers recommended revisions, and I would like to invite you to submit a revised manuscript that has considered and responded to the reviewers' points.

I do not agree with Reviewer #2's comments about your 'confusion' as to evolutionary past vs. present, nor their claim the Zietsch et al. results do not bear on the T-W assumptions you laid out. (Zietsch, Walum, Lichtenstein, Verweij, & Kuja-Halkola, 2021, `https://doi.org/10.1098/rspb.2021.0304`) provided a response to the cited commentary, and this article may help to clarify the issue. I think your treatment of T-W was very nice and clear, but you may wish to briefly address this issue in your revision so as other readers don't have the same question.

Thank you, we refer to your statement in reply to the Reviewer #2.

Please prepare and submit your revised manuscript within 30 days. If you anticipate any delay, please let us know the expected resubmission date by replying to this email.

When you are ready to resubmit, please upload the following:

[1] A letter containing a detailed list of your responses to all review comments, and a description of the changes you have made in the manuscript. Please note while forming your response, if your article is accepted, you may have the opportunity to make the peer review history publicly available. The record will include editor decision letters (with reviews) and your responses to reviewer comments. If eligible, we will contact you to opt in or out.

[2] Two versions of the revised manuscript: one with either highlights or tracked changes denoting where the text has been changed; the other a clean version (uploaded as the manuscript file).

Important additional instructions are given below your reviewer comments.

Thank you again for your submission to our journal. We hope that our editorial process has been constructive so far, and we welcome your feedback at any time. Please don't hesitate to contact us if you have any questions or comments.

Reviwer #1

The authors explored the factors that underlie deviations in the human sex ratio in humans from Sweden and the United States of America. The authors used a large dataset (> 150 million people) to discover that increased levels of a number of pollutants affect human sex ratios at birth. These pollutants could induce higher or lower sex ratios at birth, depending on the pollutant. It is a well written paper on an interesting topic and deserving of publication in PLOS Computational Biology. I only have some very minor comments.

1. The analyses are complicated. Why did you choose a bayesian approach? I'm not critical of the statistic approach taken. However, a justification for the statistical approach would be great to see in the methods.

   A frequentist analysis and a Bayesian one with weak priors generate nearly identical results, so our Bayesian choice did not bias results in any way. For practical reasons (availability of well-tested framework) we used mathematical machinery that is best fit for datasets with hierarchical/multilevel geographical structures, which provide generative models over the data.

2. There are quite a few parameters in your model. Could overparameterization be an issue here? Granted, the sample size is very large.

   In general, overparameterization in contemporary statistics is encountered in neural networks or Bayesian nonparametrics (e.g. Gaussian process, Dirichlet process), designed to have more parameters than data points for the purpose of overcoming unfavourable landscapes in optimization [cf. (Buhai, Halpern, Kim, Risteski, & Sontag, 2020; Kim, Dyer, & Rush, 2019)]. This is not the case in our regression analysis with 3100 counties (data points) and at most 56 parameters (50 for intercept/state-level random effects + 6 for slope).

3. Could you please provide the R code used to analyse this data

   The complete code is availavle at `https://github.com/yananlong/SRB`.

4. Could you also assess mean income across the populations in your models or another socioeconomic measure? Or can you justify why that is unnecessary or irrelevant?

There are several socioeconomic variables (such as median household cost, `med_hh_value`, median household income, `med_hh_inc`, and percent of persons living at less than poverty level, `pct_pers_lt_pov`) in the EQI dataset (see Table S11). None of them turned out to be statistically significant (see Tables 3 and 4 for the significant factors).

Reviwer #2

The authors present analyses of seasonal, social, and environmental influences on the sex ratio at birth. Their analyses are based upon two large databases (IBM Health and Sweden). Each contains about three million live births. They report that there is no influence of season on the sex ratio at birth. They also report significant associations between the sex ratio at birth and the level of various social "factors" (e.g., traffic fatality rate) and environmental factors (pollutants). The data on social factors comes from several sources, including US NOAA, EPA See Table S11 for list of factors.

The core of the statistical analyses is multilevel Bayesian logistic regression with random effects. The analyses appear to be performed correctly.

I have a several concerns about this manuscript.

1. The authors do not appear to understand some of the literature that they cite. For example, they write (p. 2):

   > Because human male gametes bearing X or Y chromosomes are equally frequent (being produced by meiosis symmetrically partitioning two sex chromosomes), and because ova bear only X chromosomes, one would expect a sex ratio at conception of exactly $\frac{1}{2}$.

   and they cite (Fisher, 1930) for this claim. Fisher's treatment of the evolution of the sex ratio contains no mention of sex chromosomes, equal segregation, and certainly does not involve a claim of the sex ratio at conception being or expected to be $\frac{1}{2}$. In fact, he wrote (p. 159):

   > [The attainment of the sex ratio of the equal investment equilibrium via differential mortality of males] is brought about by a somewhat larger inequality in the sex ratio at conception.

   There are many articles that could be correctly cited for the claim that one would expect an even sex ratio at conception (see (Orzack et al., 2015) for citations in which this claim is made.)

   We thank the reviewer for pointing out this important fact. We changed citation for this claim to (Boklage, 2005).

2. The authors discuss their results and how they are related to the Trivers–Willard hypothesis (TWH) (pp. 11–13). They conclude (p. 12):

   > One key ramification of the above analysis is that the TWH cannot provide a comprehensive account of the range of exogenous factors associated with SRB variation under the kind of circumstances present in our study.

I am skeptical as to the relevance of the TWH to human populations (and those of other species) but the authors' conclusion is not anchored in the specifics of their results. What are needed are specific analyses of these data that bear on the predictions of the TWH. In this context, the authors mention the study of (Zietsch, Walum, Lichtenstein, Verweij, & Kuja-Halkola, 2020) and claim that:

> In particular, Zietsch et al. have demonstrated that there exists neither within-individual SRB auto-correlation (contra Assumption A1) nor similarity in the SRB for children of siblings (contra Assumption A2).

Their study contains no analyses that bear directly on these assumptions as defined by the present authors (p. 11):

> Assumption A1. The condition of a mother during parental investment is correlated with the condition of her offspring; in other words, mothers in better conditions have offspring that will be in better conditions.
>
> Assumption A2. The condition of the offspring persists after parental investment ends, and is positively correlated with the offspring's reproductive success.

In this context, it also appears that the authors have confused the evolutionary past with the evolutionary present. The (Zietsch et al., 2020) results and those of others do suggest that there is little genetic variation for the sex ratio in human populations. Beyond that, they do not necessarily imply anything about the past influence of the selective process described by the TWH (cf. (Orzack & Hardy, 2021)). The current human sex ratio may reflect the past influence of the TWH dynamic even if that dynamic does not operate currently. That said, while I think that its realized past influence is likely negligible, it is important to note that opinions differ. At minimum, the authors need to do a better job of marshaling evidence for their claim and addressing the claims that the TWH is an important influence of human sex ratios (cf. Navara (2018)).

We made an effort to clarify this point in the revised manuscript to avoid further confusion. (Please also see the Editor's comment on this issue, quoted above.)

Please note that in the Invited Reply to the Comment article cited above [(Orzack & Hardy, 2021)], the authors explained that (Zietsch et al., 2021, §5, p. 2, emphasis added):

> One of the most popular theories regarding human offspring sex ratio is the Trivers–Willard hypothesis, which proposes that parents adaptively adjust their offspring sex ratio according to their condition. [...] *If the offspring sex ratio is calibrated to a variable that is to some degree heritable (or just consistent within individuals over time), then offspring sex ratio must itself be to some degree heritable (or consistent within individuals over time).* Given that human quantitative traits are invariably heritable to some extent (and at the very least show some consistency within individuals), our data are incompatible with the Trivers–Willard hypothesis as it has been applied to humans. Fisher's principle and the Trivers–Willard hypothesis are by far the dominant

> evolutionary explanations for human offspring sex ratio, so if neither can explain the observations, then we maintain that sex ratio theory needs a rethink, at least as it applies to humans.

which echoes the original article (Zietsch et al., 2020, §4, p. 5, emphasis added):

> Our results also rule out the possibility that offspring sex ratios are adaptively calibrated to individuals' heritable traits. Certain interpretations of the Trivers–Willard effect propose that parents who possess any heritable trait that disproportionately benefits the fitness of one sex will bias their offspring sex ratio towards that sex. [...] Our findings are incompatible with the basic effect: *if offspring sex ratio was calibrated to heritable traits, then it would necessarily be heritable to some degree as well.*

Moreover, it is important to observe that the results of (Zietsch et al., 2020) were quite general regarding *any* heritable traits that would impact SRB. As we explained in our original submission (p. 12, emphasis added):

> such evidence also places other adaptive (i.e. via *heritable* sexual selection) theories explaining SRB variations, such as adaptive versions of hormonal hypothesis (James, 2008), maternal dominance hypothesis (Grant, 2003, 2007) and the Bruce effect (Catalano et al., 2018) in the same predicament.

As for the distinction between evolutionary past vs evolutionary present, Orzack and Hardy wrote, in the context of Fisher's principle (§1, p. 1):

> the sex ratio of *H. sapiens* would be at least in part a result of past evolution, instead of being entirely a result of the current evolution in human populations, and indeed this potential influence of past evolution is mentioned by (Zietsch et al., 2020, p. 7). Consideration of the influence of such 'phylogenetic inertia' [...] is rare among analyses that attempt to compare the predictions of sex allocation theory to data from humans and other vertebrates and can render their conclusions ambiguous.

To this Zietsch et al. replied (§3, p. 2):

> [F]or Fisher's principle to be a useful explanation, it must be able to explain something. The basic properties of human offspring sex ratio, which can be observed in our original paper, are that it is roughly even on average but varies across individuals in a way that indicates no variation in the underlying probability of having a son versus a daughter (i.e. no heritable variation nor any pattern in the sex of subsequent offspring within individuals). Fisher's principle cannot explain these features. Random Mendelian sex determination can explain them, though the ultimate origin of this proximate mechanism is unknown. Fisher's principle, whether or not it ever occurred, does not provide an ultimate explanation. There is no logical connection between the state in which Fisher's principle could operate (heritable offspring sex ratio) and the state we observe (zero heritability).

The upshot is that (§1, p. 1, emphasis added)

> [E]limination of genetic variation is not expected under Fisher's principle. Therefore, even if offspring sex ratio were heritable at some time in our evolutionary history (a possibility we mentioned, p. 7), Fisher's principle could not explain how it went from heritable to not heritable. That is, Fisher's principle cannot explain variation in human offspring sex ratio as we observe it. *When theory cannot explain data, the theory needs rethinking*.

3. Finally, the authors do not correctly represent some of the prior literature pertaining to environmental influences on the human sex ratio. They write (p. 8)

> Using the US dataset, we were able to validate the findings of a number of previous studies regarding the association between the SRB and exogenous factors (Table 3). Specifically, our data suggests that PCBs (polychlorinated biphenyls), aluminium (Al) in air, chromium (Cr) in water and total mercury (Mg) quantity drive the SRB up, while lead (Pb) in soil appears to be associated with a decreased SRB.

This statement implies, for example, that the influence of PCBs on the human sex ratio is resolved. This implication is incorrect for two reasons. The first is that there are conflicting results in the literature, with some showing an increased sex ratio with PCB exposure and others the opposite [Hertz-Picciotto et al. (2008); Leijs et al. (2014); Mackenzie, Lockridge, and Keith (2005); Nieminen, Lehtiniemi, Huusko, Vähäkangas, and Rautio (2013); Terrell et al. (2009); Terrell, Hartnett, and Marcus (2011); Vartiainen, Kartovaara, and Tuomisto (1999); Weisskopf, Anderson, and Hanrahan (2003)]. The second reason is that, if anything, a common understanding is that, in fact, PCB exposure is associated with a decrease in sex ratio, not an increase as claimed by the authors. At minimum, the authors need to acknowledge these heterogeneous results and how their results relate to them. Better would be an attempt to explain if and how their results help resolve the discrepancies among studies.

We thank the reviewer for alerting us to the ongoing debate around this issue. The relevant passage has been altered accordingly.

References

Boklage, C. E. (2005). The epigenetic environment: secondary sex ratio depends on differential survival in embryogenesis. *Human Reproduction*, *20*(3), 583–587.

Buhai, R.-D., Halpern, Y., Kim, Y., Risteski, A., & Sontag, D. (2020). Empirical study of the benefits of overparameterization in learning latent variable models. In *International conference on machine learning* (pp. 1211–1219).

Catalano, R., Gemmill, A., Casey, J., Karasek, D., Stewart, H., & Saxton, K. (2018). Separating the bruce and trivers-willard effects in theory and in human data. *American Journal of Human Biology*, *30*(2), e23074.

Fisher, R. A. (1930). The genetical theory of natural selection.

Grant, V. J. (2003). The maternal dominance hypothesis: questioning trivers and willard. *Evolutionary Psychology*, *1*(1), 147470490300100106.

Grant, V. J. (2007). Could maternal testosterone levels govern mammalian sex ratio deviations? *Journal of Theoretical Biology*, *246*(4), 708–719.

Hertz-Picciotto, I., Jusko, T. A., Willman, E. J., Baker, R. J., Keller, J. A., Teplin, S. W., & Charles, M. J. (2008). A cohort study of in utero polychlorinated biphenyl (pcb) exposures in relation to secondary sex ratio. *Environmental Health*, *7*(1), 1–8.

James, W. H. (2008). Evidence that mammalian sex ratios at birth are partially controlled by parental hormone levels around the time of conception. *The Journal of endocrinology*, *198*(1), 3–15.

Kim, Y., Dyer, C., & Rush, A. M. (2019). Compound probabilistic context-free grammars for grammar induction. *arXiv preprint arXiv:1906.10225*.

Leijs, M., Van der Linden, L., Koppe, J., Olie, K., Van Aalderen, W., & Ten Tusscher, G. (2014). The influence of perinatal and current dioxin and pcb exposure on reproductive parameters (sex-ratio, menstrual cycle characteristics, endometriosis, semen quality, and prematurity): a review. *Biomonitoring*, *1*(1).

Mackenzie, C. A., Lockridge, A., & Keith, M. (2005). Declining sex ratio in a first nation community. *Environmental health perspectives*, *113*(10), 1295–1298.

Navara, K. J. (2018). *Choosing sexes: mechanisms and adaptive patterns of sex allocation in vertebrates*. Springer.

Nieminen, P., Lehtiniemi, H., Huusko, A., Vähäkangas, K., & Rautio, A. (2013). Polychlorinated biphenyls (pcbs) in relation to secondary sex ratio–a systematic review of published studies. *Chemosphere*, *91*(2), 131–138.

Orzack, S. H., & Hardy, I. C. (2021). Does the lack of heritability of human sex ratios require a rethink of sex ratio theory? no: a comment on zietsch et al. 2020. *Proceedings of the Royal Society B*, *288*(1947), 20202638.

Orzack, S. H., Stubblefield, J. W., Akmaev, V. R., Colls, P., Munné, S., Scholl, T., ... Zuckerman, J. E. (2015). The human sex ratio from conception to birth. *Proceedings of the National Academy of Sciences*, *112*(16), E2102–E2111.

Terrell, M. L., Berzen, A. K., Small, C. M., Cameron, L. L., Wirth, J. J., & Marcus, M. (2009). A cohort study of the association between secondary sex ratio and parental exposure to polybrominated biphenyl (pbb) and polychlorinated biphenyl (pcb). *Environmental Health*, *8*(1), 1–12.

Terrell, M. L., Hartnett, K. P., & Marcus, M. (2011). Can environmental or occupational hazards alter the sex ratio at birth? a systematic review. *Emerging health threats journal*, *4*(1), 7109.

Vartiainen, T., Kartovaara, L., & Tuomisto, J. (1999). Environmental chemicals and changes in sex ratio: analysis over 250 years in finland. *Environmental Health Perspectives*, *107*(10), 813–815.

Weisskopf, M. G., Anderson, H. A., & Hanrahan, L. P. (2003). Decreased sex ratio following maternal exposure to polychlorinated biphenyls from contaminated great lakes sport-caught fish: a retrospective cohort study. *Environmental Health*, *2*(1), 1–14.

Zietsch, B. P., Walum, H., Lichtenstein, P., Verweij, K. J., & Kuja-Halkola, R. (2020). No genetic contribution to variation in human offspring sex ratio: a total population study of 4.7 million births. *Proceedings of the Royal Society B*, *287*(1921), 20192849.

Zietsch, B. P., Walum, H., Lichtenstein, P., Verweij, K. J., & Kuja-Halkola, R. (2021). When theory cannot explain data, the theory needs rethinking. invited replies to: Orzack SH, Hardy ICW. 2021, and Lehtonen J. 2021.

Proceedings of the Royal Society B, *288*(1947), 20210304.
