# Supplementary material for: Observable variations in human sex ratio at birth
Source: PLoS Comput Biol. 2021 Dec 2;17(12):e1009586. doi: 10.1371/journal.pcbi.1009586 (PMC8638995; doi:10.1371/journal.pcbi.1009586)
Supplement: Attachment — Submitted filename: SRB_PLoS_comp_bio_response3.pdf [file pcbi.1009586.s003.pdf]

Responses to Reviewers Version 2

Editor's comments

Only minor issues remain, and these do not warrant another round of review.

1. "In other words, if SRB is ever influenced by some factor(s) at least partially inheritable, then SRB itself would have to be heritable as well which the results from Zietsch et al. rule out."

   "Inheritable" has a different meaning from "heritable" — heritable is the appropriate word here.

   We thank the editor for pointing out the typo.

2. "Thus, our results are better interpreted as supporting the intrinsic randomness of the SRB and/or the dependence of the SRB on non-adapative (e.g. socio-cultural [71, 77, Ch. 12]), causal factors, possibly including those common to both changes in the SRB and associated exogenous factors."

   It doesn't quite make sense to say that your results, which involve associations with other variables, support the intrinsic randomness of SRB. If SRB was truly random, then it wouldn't be associated with other variables. Rephrase, distinguishing 1) the strong influence of random Mendelian randomisation from 2) the SRB itself, which is not quite random, as these results show.

   We thank the editor for this helpful conceptual clarification. It seems to us that by "random Mendelian randomisation" it is meant "random Mendelian segregation". Mendelian randomization is a statistical technique, not a natural phenomenon.

3. The authors should go through the text carefully checking for grammatical issues, especially the placement of commas. Often there were commas where there shouldn't be. Also check spelling (e.g. non-adapative in the above quote).

   We thank the editor for alerting us to the grammatical issues.

Formatting comments

Following modifications were made:

1. We note that the following authors have not yet confirmed their authorship on this manuscript: Henrik Larsson

   At this point this request is not urgent; we have re-sent their co-author confirmation email containing an auto-confirmation link for them to click, and we will reach out to them at a later stage if we don't hear from them. We wanted to mention this in case you are aware of them experiencing technical issues with confirming authorship via the link. If so, please do ask them to contact us directly via email and we'd be happy to assist.

We contacted Professor Larsson, and he informed us that the authorship was confirmed.

2. We note that your supplementary files are duplicated on your submission. Please remove any unnecessary or old files from your revision, and make sure that only those relevant to the current version of the manuscript are included.

Thank you, corrected.

3. Please provide a detailed Financial Disclosure statement. This is published with the article, therefore should be completed in full sentences and contain the exact wording you wish to be published.

   Please clarify all sources of funding (financial or material support) for your study. List the grants (with grant number) or organizations (with url) that supported your study, including funding received from your institution.

   Please ensure you include your funder's role statement "The funders had no role in study design, data collection and analysis, decision to publish, or preparation of the manuscript." at the end of your amended statement.

Done. The following was added: "This work was funded by the DARPA Big Mechanism program under ARO contract W911NF1410333, by National Institutes of Health grants R01HL122712, 1P50MH094267, and U01HL108634-01, and by a gift from Liz and Kent Dauten. Additional support came from King Abdullah University of Science and Technology (KAUST), awards number FCS/1/4102-02-01, FCC/1/1976-26-01, REI/1/0018-01-01, and REI/1/4473-01-01. The funders had no role in study design, data collection and analysis, decision to publish, or preparation of the manuscript."

4. Thank you for stating "Data and code made available on GitHub." Please note that your Data Availability Statement is currently missing the DOI/accession number of each dataset OR a direct link to access each database. If your manuscript is accepted for publication, you will be asked to provide these details on a very short timeline. We therefore suggest that you provide this information now, though we will not hold up the peer review process if you are unable.

Corrected. This is how it reads now: "Aggregate data associated with described analyses and R scripts used in this analysis are available at `https://github.com/yananlong/SRB`. In particular, `https://git.io/Jg5zx` for the list of counties with neighbours, `https://git.io/Jg5iy` for the number of male and female

newborns plus the exogenous factors derived from the EQI dataset for each county, and https://git.io/Jg5MJ for the county, sex and birthday for each mother–newborn pair.”
